# Discovering biclusters in gene expression data based on high-dimensional linear geometries

**DOI:** 10.1186/1471-2105-9-209

**Published:** 2008-04-23

**Authors:** Xiangchao Gan, Alan Wee-Chung Liew, Hong Yan

**Affiliations:** 1 Department of Computer Science, King's College London, UK; 2 School of Information & Communication Technology, Griffith University, Queensland, Australia; 3 Department of Electronic Engineering, City University of Hong Kong, Hong Kong, China; 4 School of Electrical & Information Engineering, University of Sydney, NSW 2006, Australia

## Abstract

**Background:**

In DNA microarray experiments, discovering groups of genes that share similar transcriptional characteristics is instrumental in functional annotation, tissue classification and motif identification. However, in many situations a subset of genes only exhibits consistent pattern over a subset of conditions. Conventional clustering algorithms that deal with the entire row or column in an expression matrix would therefore fail to detect these useful patterns in the data. Recently, biclustering has been proposed to detect a subset of genes exhibiting consistent pattern over a subset of conditions. However, most existing biclustering algorithms are based on searching for sub-matrices within a data matrix by optimizing certain heuristically defined merit functions. Moreover, most of these algorithms can only detect a restricted set of bicluster patterns.

**Results:**

In this paper, we present a novel geometric perspective for the biclustering problem. The biclustering process is interpreted as the detection of linear geometries in a high dimensional data space. Such a new perspective views biclusters with different patterns as hyperplanes in a high dimensional space, and allows us to handle different types of linear patterns simultaneously by matching a specific set of linear geometries. This geometric viewpoint also inspires us to propose a generic bicluster pattern, i.e. the linear coherent model that unifies the seemingly incompatible additive and multiplicative bicluster models. As a particular realization of our framework, we have implemented a Hough transform-based hyperplane detection algorithm. The experimental results on human lymphoma gene expression dataset show that our algorithm can find biologically significant subsets of genes.

**Conclusion:**

We have proposed a novel geometric interpretation of the biclustering problem. We have shown that many common types of bicluster are just different spatial arrangements of hyperplanes in a high dimensional data space. An implementation of the geometric framework using the Fast Hough transform for hyperplane detection can be used to discover biologically significant subsets of genes under subsets of conditions for microarray data analysis.

## Background

In DNA microarray experiments, discovering groups of genes that share similar transcriptional characteristics is instrumental in functional annotation, tissue classification and motif identification [1,2]. In many situations, an interesting cellular process is active only under a subset of conditions, or a single gene may participate in multiple pathways that may or may not be co-active under all conditions [3,4]. In addition, the data to be analyzed often include many heterogeneous conditions from many experiments. In these instances, it is often unrealistic to require that related genes behave similarly across all measured conditions and conventional clustering algorithms, such as the k-means and hierarchical clustering algorithms [5,6] and the self-organizing map [7], often cannot produce a satisfactory solution.

**Figure 1:**
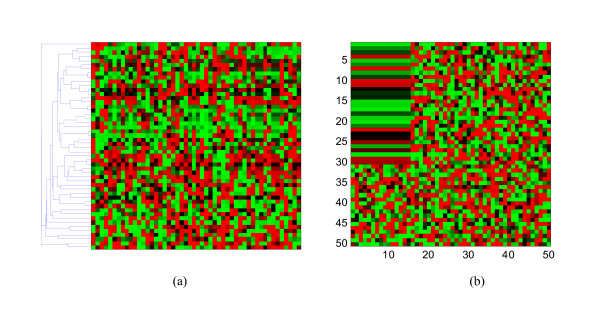
**An illustrative example where conventional clustering fails but biclustering works:** (a) A data matrix, which appears random visually even after hierarchical clustering. (b) A hidden pattern embedded in the data would be uncovered if we permute the rows or columns appropriately.

When a subset of genes shares similar transcriptional characteristics only across a subset of measures, the conventional algorithm may fail to uncover useful information between them. In Fig. 1a, we see a data matrix clustered using the hierarchical clustering algorithm, where no coherent pattern can be observed by naked eyes. However, Fig. 1b indicates that an interesting pattern actually exists within the data if we rearrange the data appropriately.

**Figure 2:**
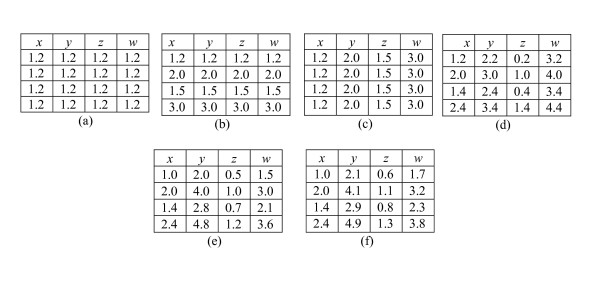
**Examples of different bicluster patterns:** (a) constant values, (b) constant rows, (c) constant columns, (d) additive coherent values, (e) multiplicative coherent values, and (f) linear coherent values.

The hidden pattern in Fig. 1b is called a bicluster. One of the criteria to evaluate a biclustering algorithm is what kind of bicluster patterns an algorithm is able to find. In this paper, we address six major classes of numerical biclusters. Fig. 2 shows different patterns that are of interest to us: (a) constant values, (b) constant rows, (c) constant columns, (d) additive coherent values, where each row or column is obtained by adding a constant to another row or column, (e) multiplicative coherent values, where each row or column is obtained by multiplying another row or column by a constant value, and (f) linear coherent values, where each column is obtained by multiplying another column by a constant value and then adding a constant. Among these patterns, the first 5 patterns have been introduced by Madeira and Oliveira [8]. Patterns (a-c) are compatible to (d) or (e), in the sense that an algorithm which can detect additive patterns can also detect constant rows/column since the latter are two special cases of the former, while (d) and (e) are mutually independent. Most existing algorithms are based on either the additive model (d) or the multiplicative model (e). The linear coherent pattern of (f) is a generalization proposed by us and subsumes all patterns in Fig. 2. These patterns can be more easily understood based on our geometric perspective introduced below.

In this work, we deal with numerical biclusters only. There are also works [9,10] that focus on biclusters containing symbolic data or the so-called coherent evolution biclusters, where the evolution (i.e., up, down, or no change) of the elements in a numerical data matrix is considered instead of the numerical values themselves. We choose to focus on the numerical data based on the following considerations. First, a numerical biclustering algorithm can be used to analyze symbolic data by assigning appropriate numerical values to the symbols. Second, many gene expression data analysis tasks, such as gene regulation network analysis, require numerical biclustering results.

### Previous work on biclustering

Throughout the paper, we use *F *∈ ℜ^*N *× *M *^to denote a gene expression data matrix with *N *genes and *M *arrays or experiment conditions. In the matrix *F*, a row *F*_*i *_∈ ℜ^1 × *M *^represents the expression of the gene *i *in *M *arrays. For simplicity, we only introduce biclustering algorithm for constant/coherent rows below, the corresponding algorithm for constant/coherent columns is similar and can be easily deduced.

Bicluster of constant values is obviously the simplest type. A bicluster of constant values can be modeled as

(1) *F*(*i*, *j*) = *u*_*IJ *_+ *ε*(*i*, *j*),

where *u*_*IJ *_is the typical value of the bicluster and *ε*(*i*,*j*) is a small perturbation. Hartigan [11] split the original matrix into a predetermined set of submatrices, and use the variance to evaluate each submatrix $VAR(I,J)=\sum_{i\in I,j\in j}F(i,j)-u_{IJ}$ to determine whether a bicluster should be accepted.

If the noise is additive, a bicluster of constant rows can be modeled as

*F*(*i*,*j*) = *u*_*IJ *_+ *f*_*i *_+ *ε*(*i*,*j*).

where *f*_*i *_is the *i*-th row offset. The straightforward method to detect a bicluster of constant row is to normalize the rows of the bicluster using the row mean. By doing so, a bicluster of constant row can be transformed into a bicluster of constant values and hence becomes detectable using algorithms for biclusters of constant values. Getz et al. [12] have developed a method based on this consideration and even extended it to detect biclusters of coherent values. However, methods based on data normalization have a dilemma: for a good normalization, we need to estimate the parameter *f*_*i *_for each row of a bicluster. However, for an accurate estimate of *f*_*i*_, we need to know the location of a bicluster, which is exactly the problem we need to solve. The noise *ε*(*i*,*j*) in the data further complicates the estimation of *f*_*i*_. Instead of relying on data normalization, Califano *et al*. [13] have developed a method to find some small biclusters first with each row satisfying

max(*F*(*i*,*j*)) - min(*F*(*i*,*j*)) <*δ*, ∀*j *∈ *J*

and then add additional rows or columns into it to produce a bicluster that is as large as possible. Sheng *et al*. [14] have assumed that the multinomial distributions for different columns in a bicluster are mutually independent and used the Gibbs sampling for parameter estimation.

A bicluster of additive coherent values with additive noise can be modeled as

(2) *F*(*i*, *j*) = *u*_*IJ *_+ *f*_*i *_+ *g*_*j *_+ *ε*(*i*, *j*).

Cheng and Church [15] are the first who applied biclustering to microarray data analysis. In their method, the mean squared residue $\frac{1}{\left|I||J\right|}\sum_{i\in I,j\in J}\varepsilon {\left(i,j\right)}^{2}$ in (2) is minimized. Cho et al. [16] have improved this mean-squared-residue based method by using the variance as the second measure. Lazzeronic and Owen [17] have introduced a plaid model and proposed the general additive model to identify biclusters of constant rows, constant columns and additive coherent values. Prelic et al. [18] have compared many biclustering algorithms using the additive model.

A bicluster of multiplicative coherent values with additive noise can be modeled as

(3) *F*(*i*, *j*) = *u*_*IJ *_× *f*_*i *_× *g*_*j *_+ *ε*(*i*, *j*)

Kluger *et al*. [19] have studied the checkerboard structure of this type of biclusters using a normalization scheme based on the above equation. Tang et al. [20] have developed a method to compute the cosine value of the angle between each normalized row vector and a predefined stable pattern and then measure the similarity between two rows or two columns. Getz et al. [12] have introduced the Couple Two-Way Clustering by repeatedly performing one-way clustering on the rows and columns of the data matrix.

Madeira and Oliveira [8] are the first to classify many existing numerical biclustering algorithms systematically based on the additive and multiplicative bicluster models. It should be pointed out that some symbolic, coherent evolution or numerical biclusters, such as those produced by cMonkey [9], SAMBA [10] and some statistical criteria, cannot be classified as additive or multiplicative patterns directly. For example, in cMonkey, additional information besides the usual gene expression value, such as motif co-occurrence and association network relationships, are taken into account. Moreover, cMonkey attempts to ensure that a greater percentage of genes that are observed in the data set are included in at least one cluster, while reducing redundancy between overlapping biclusters and maximizing the number of conditions that are included in each bicluster. These features cannot be modeled directly using the additive and multiplicative coherent patterns.

Although the classification into additive or multiplicative patterns is not perfect, it is nevertheless applicable to many existing biclustering algorithms, which can all be formulated using the general linear model proposed in this paper. In fact, in most biclustering algorithms that deal with expression values only, the underlying theme is the coherency in expression values within the biclusters. Our general linear model of Fig. 2(f) therefore conveniently captures the zero and first order coherent relationships within a bicluster.

### A high-dimensional geometric method for biclustering

As pointed out in [8], existing approaches are often based on searching for sub-matrices within a data matrix by optimizing certain heuristically defined merit functions. Obviously, the form of the merit function depends greatly on the bicluster pattern to be uncovered. In these methods, when the data contain different types of biclusters, multiple merit functions or different data normalizations or transformations are needed. This often results in a high computational complexity and the optimization procedure is NP-hard in general can be easily trapped at a local optimal point.

In this paper, we extend our previous work [21] and present a novel perspective for biclustering problem through a geometric interpretation. Such a new perspective allows us to regard biclusters with different coherent patterns as hyperplanes in a high dimensional space, and facilitates the use of any generic plane finding algorithm for detecting them. The geometric viewpoint of our approach provides a unified framework to handle different types of linear patterns simultaneously by matching a specific set of linear geometries. It also reveals the existence of the general linear model, which can unify the additive and the multiplicative models. As a particular realization of our framework, we implemented a Hough Transform-based hyperplane detection algorithm. The experimental results on human lymphoma gene expression dataset show that our algorithm is highly effective for gene expression data biclustering.

## Results

We tested our algorithm using synthetic dataset and human lymphoma dataset. For synthetic dataset, we use a test model proposed in [14], but deal with both additive and multiplicative biclusters. In the Gibbs sampling method [14], only additive biclusters are used. For human lymphoma dataset, we detect biclusters based on additive, multiplicative and general linear models, and investigate whether the detected biclusters are biological meaningful. Our experiments show that the proposed linear coherent model can produce biologically significant groups enriched by the genes in biclusters.

### Synthetic dataset

**Figure 3:**
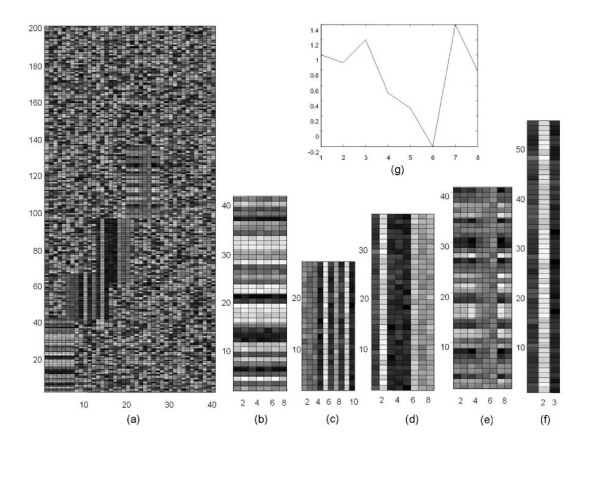
**A synthetic dataset with multiple overlapping biclusters of different patterns and the biclusters extracted using the proposed method.** (a) The data matrix before random row and column permutation, (b) bicluster 1 of constant rows, (c) bicluster 2 of constant columns, (d) bicluster 3 of constant columns, (e) bicluster 4 of multiplicative coherent values, (f) the extra bicluster extracted by the proposed method, and (g) the multiplicative coefficients of each row in bicluster 4.

We generated a synthetic dataset containing four overlapping biclusters of constant columns, constant rows, and multiplicative coherent values, and tested the ability of our approach to detect these patterns simultaneously. To test noise resistance of our method, we embedded the biclusters into a noisy background generated by a uniform distribution *U*(-5, 5). Gaussian noise with variance of 0.3 was used to degrade the biclusters. The dataset has 200 rows by 40 columns, and the embedded biclusters have the following sizes (rows × columns): 40 × 7 for Bicluster 1 of constant row, 25 × 10 for Bicluster 2 of constant column, and 35 × 8 for Bicluster 3 of constant column, 40 × 8 for Bicluster 4 of multiplicative coherent values with the multiplicative coefficients for each row given in Fig. 3g. As shown in the main plot of Fig. 3a, Bicluster 1 overlaps with Bicluster 2 in two columns, and Bicluster 3 overlaps with Bicluster 2 in five rows and three columns. Random row and column permutations are then performed in Fig. 3a to obtain the final test dataset.

In this experiment, the three biclusters contain additive coherent values, and both the Gibbs sampling method [14] and our algorithm can identify all of them, but with different accuracies. The Gibbs sampling method misses 2 genes in bicluster 2 and 4 genes in bicluster 3, whereas our algorithm detects all genes perfectly (Fig. 3). Interestingly, a new bicluster with 3 conditions and 60 rows was also reported by our method (Fig. 3f). This bicluster is located in the overlap region of biclusters 2 and 3 and comprises of last three columns of bicluster 2 and first three columns of bicluster 3 and all rows of the two biclusters. Although unexpected, this is a reasonable result since the extra bicluster detected is a valid bicluster by itself. In contrast, the Gibbs sampling method fails to detect this extra, but valid bicluster. The detection of this new bicluster further shows the efficacy of our algorithm in handling overlapping biclusters.

### Biological Data: Human Lymphoma Dataset

We apply our algorithm to the lymphoma dataset [22]. This dataset is characterized by well defined expression patterns differentiating three types of lymphoma: diffuse large B-cell lymphoma, chronic lymphocytic leukaemia and follicular lymphoma. The dataset consists of expression data from 128 Lymphochip microarrays for 4026 genes in 96 normal and malignant lymphocyte samples. Missing values in the dataset are imputed using POCSimpute [23].

We compare our algorithm with six existing algorithms, i.e., OPSM [24], Bimax [18], Iterative Signature Algorithm, ISA [25], SAMBA [10], Cheng and Church's algorithm, CC [15] and xMotif [26], using the procedure proposed by Prelic et al. [18]. Since most existing numerical biclustering algorithms do not detect biclusters with general linear coherent values, we only compare the performance for the additive model. Similar to the validation method proposed by Tanay et al. [10], we investigate whether the gene groups produced by different algorithms show significant enrichment with respect to a specific Gene Ontology (GO) annotation. We know that biclustering algorithms aim to classify the genes involved in the same Molecular Function or Biological Process into a group, so a better biclustering algorithm can find more or larger groups that show significant enrichment. Specifically, in our experiment, biclusters are evaluated by computing the hyper-geometric functional enrichment score [27] based on the GO Biological Process annotations, and the resulting scores are adjusted for multiple testing using the Westfall and Young procedure [27,28].

**Figure 4:**
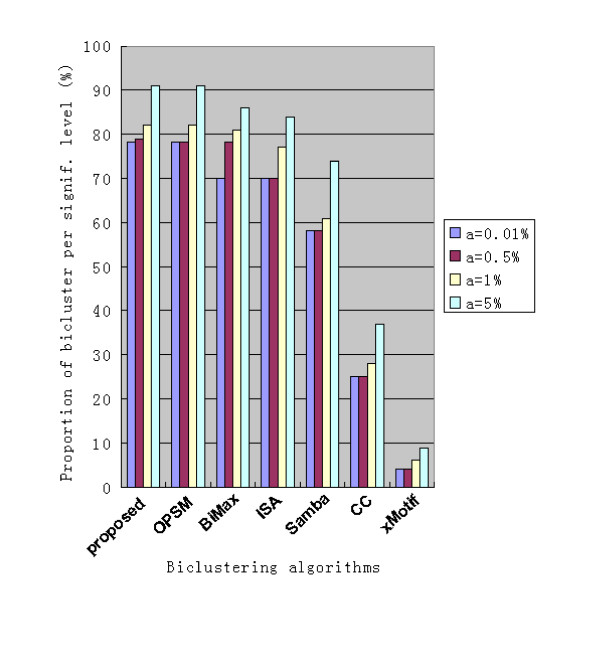
**Proportion of biclusters significantly enriched by a GO Biological Process category for the six selected biclustering methods.** The columns are grouped method-wise, and different bars within a group represent the results obtained for five different significance levels *α*.

The histogram in Fig. 4 presents the proportion of biclusters produced by each method for which one or more GO categories are overrepresented at different levels of significance. Best results are obtained from OPSM and the proposed algorithm. Our algorithm is very competitive even when we only consider additive biclusters.

**Figure 5:**
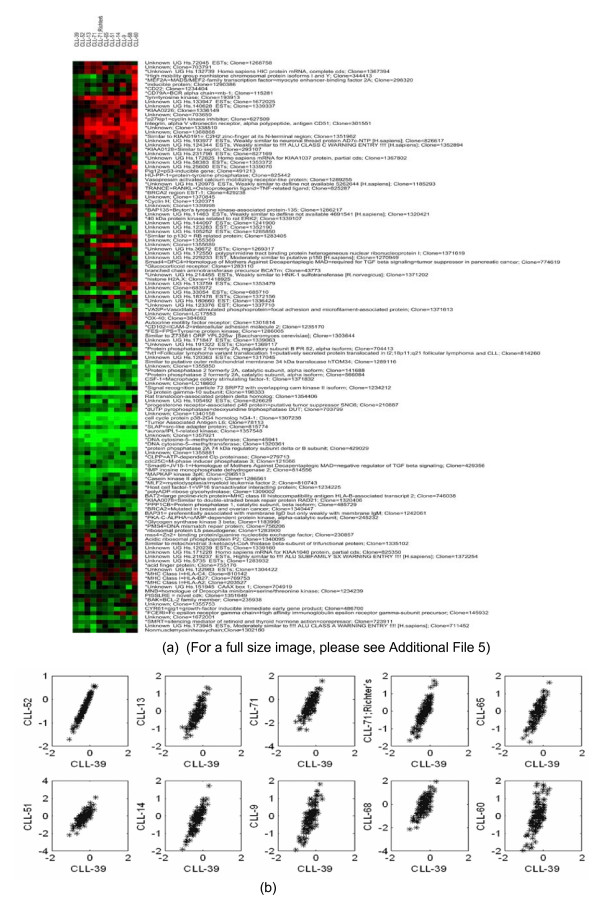
**Biclusters detected in the lymphoma dataset.** (a) A bicluster of linear coherent values detected by our algorithm (for the full size image, please see Additional File 5), (b) scatter plots showing the linearity amongst the columns in this bicluster.

**Figure 6:**
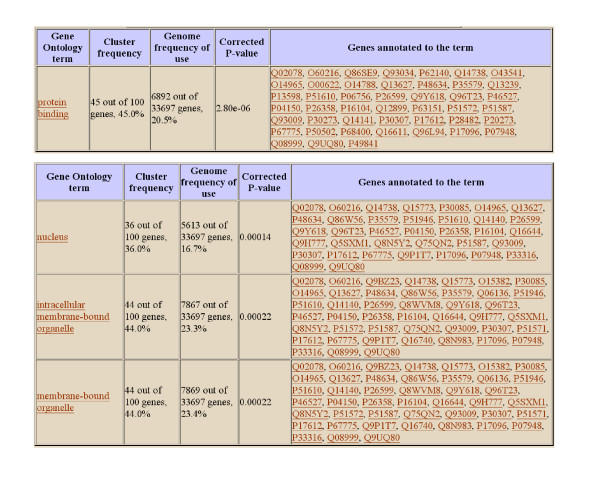
**The GO-based evaluation for the bicluster of Fig. 5a using the GOTermFinder.** The upper table is from the biological process ontology; the middle table is from the molecular function ontology; and the lower table is from the cellular component ontology.

Our method is also capable of detecting biclusters with general linear coherent values. Fig. 5a shows one of these biclusters detected in the lymphoma dataset. The linearity amongst the columns in this bicluster is verified using the scatter plots in Fig. 5b and a good fit can be observed. By defining the column of this bicluster as *F*_0_, *F*_1_, ..., *F*_10_, the pattern of this bicluster can be expressed as *F*_0 _= 0.57*F*_1 _- 0.08 = 0.38*F*_2 _- 0.24 = 0.27F_3 _- 0.15 = 0.36*F*_4 _- 0.26 = 0.36*F*_5 _- 0.27 = 0.30*F*_6 _- 0.25 = 0.37*F*_7 _- 0.22 = 0.28*F*_8 _- 0.27 = 0.27*F*_9 _- 0.28 = 0.22*F*_10 _- 0.29. The detailed results from the GOTermFinder at significance level of 5% are provided in Fig. 6. The result from the GO analysis shows that these linear coherent biclusters are indeed biological meaningful.

In Additional File 1, we provide the algorithmic parameters used in the experiment for the lymphoma dataset. In our experiment, more than 600 biclusters are detected. In Additional File 2, we provide a list of all biclusters with 1 showing corresponding genes/arrays covered by the bicluster while 0 is the contrary. In Additional File 3, we selected 6 biclusters as an example for GO annotation. All the biclusters with full data are given in Additional File 4. The full-sized image of Fig. 5a is shown in Additional File 5.

## Conclusion

We analyzed the different type of numerical biclusters and proposed a general linear coherent bicluster model that effectively captures the zero and first order coherent relationships within a bicluster. Then, we presented a novel interpretation of the biclustering problem in terms of the geometric distributions of data points in a high dimensional data space. In this perspective, the biclustering problem becomes that of detecting structures of known linear geometries, i.e., hyperplanes, in the high dimensional data space. We have shown that many common types of bicluster are just different spatial arrangements of the hyperplanes in the high dimensional data space. This novel perspective allows us to perform biclustering geometrically using a hyperplane detection algorithm. The experiment results on both synthetic and real gene expression datasets have demonstrated that our algorithm is very effective.

## Method

Although the six patterns in Fig. 2 appear to be substantially different from each other, if we treat each measurement (column) as a variable in the 4D space [*x*, *y*, *z*, *w*] and each object (row) as a point in the 4D space, the six pattern in Figs. 2(a) to 2(f) would correspond to the following six geometric structures respectively: (a) a cluster at a single point with coordinate [*x*, *y*, *z*, *w*] = [1.2, 1.2, 1.2, 1.2], (b) a cluster defined by the lines *x *= *y *= *z *= *w*, (c) a cluster at a single point with coordinate [*x*, *y*, *z*, *w*] = [1.2, 2.0, 1.5, 3.0], (d) a cluster defined by the lines *x *= *y *- 1 = *z *+ 1 = *w *- 2, (e) a cluster defined by the lines *x *= 0.5*y *= 2*z *= 2*w*/3, and (f) a cluster defined by the lines *x *= 0.5(*y *- 0.1) = 2(*z *- 0.1) = 2(*w *- 0.2)/3. Each object (row) in a cluster is a point lying on one of these points or lines.

**Figure 7:**
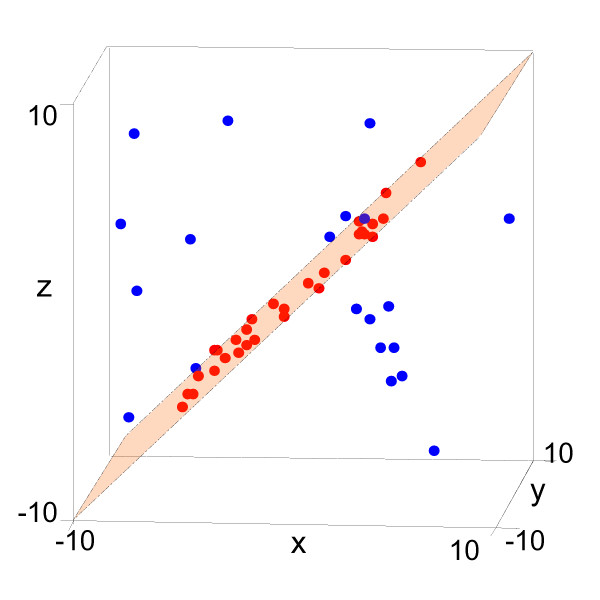
**If we visualize the data in Fig. 1a in a high-dimensional space, the hidden pattern stands out.** Due to the difficulties in visualizing data beyond 3D, we only select columns 32, 41 and 45 in Fig. 1a to form a new data matrix with a 2-column bicluster embedded inside. In this figure, there exists an obvious plane, which provides clues about the hidden bicluster in the data.

When a pattern is embedded in a larger data matrix with extra measurements, i.e., a bicluster that covers only part of the measurements in the data, the points or lines defined by the bicluster would sweep out a hyperplane in a high dimensional data space. Assume that we have a three-measurement experiment with the measurements denoted by *x*, *y*, and *z*. If a bicluster covers measurements x and z, then there exists a plane where all data points in the bicluster would lie on. The plane is defined by:

(4) *β*_0 _+ *β*_1_*x *+ *β*_3_*z *= 0

where *β*_*i*_, (*i *= 0, 1, 3) are constants and *β*_2_*y *is omitted since *β*_2 _= 0. The coordinates that appeared in Eq. (4) denote the measurements the bicluster covers, and the points on the plane denote the objects or genes in that bicluster. In Fig. 7, an example of such a plane is shown. We select 3 columns from the data matrix of Fig. 1a and form a new data matrix with a 2-column bicluster embedded inside. The new data matrix is then plotted in a 3D space. We can see that there exists an obvious plane, which provides clues about the hidden bicluster in the data. The linear model has been used in the clustering method OSCAR developed by Bondell and Reich [29]. A major difference between OSCAR and our algorithm is that OSCAR carries out clustering or classification in one direction only, while our algorithm performs biclustering or simultaneous clustering in both row and column directions of the data matrix.

**Figure 8:**
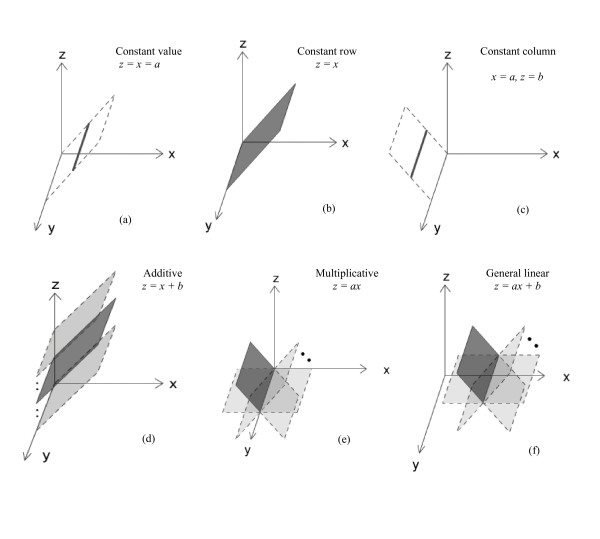
**Different geometries (lines or planes) in the 3D data space for corresponding bicluster patterns.** In each table, the shaded columns are covered by a bicluster. (a) A bicluster with constant values: represented by one of the lines that are parallel to the *y*-axis and lie in the plane *x *= *z *(the T-plane), (b) a bicluster with constant rows: represented by the T-plane, (c) a bicluster with constant columns: represented by one of the lines parallel to the *y*-axis, (d) a bicluster with additive coherent values: represented by one of the planes parallel to the T-plane, (e) a bicluster with multiplicative coherent values: represented by one of the planes that include the *y*-axis, and (f) a bicluster with linear coherent values: represented by one of the planes that are parallel to the *y*-axis.

In general, different bicluster patterns discussed above can be uniquely defined by specific geometric structures (lines, planes or hyperplanes) in a high dimensional data space. In a 3D space, if we denote the three measurements as *x*, *y *and *z *respectively, and assume a bicluster covers *x *and *z *only, we can generate 3D geometric views for different patterns as shown in Fig. 8. When the dimension of the data space is more than three, it becomes difficult to visualize the data points, but the geometric structures are still similar. In addition, this geometric perspective provides valuable insight to the property of biclusters. For example, current algorithms often deal with additive and multiplicative models separately. When we analyze the 3D geometries of these two types of biclusters (Figs. 8d and 8e), it is obvious that there are geometries (Fig. 8f) covering both of them. These new kind of geometries denote a new type of biclusters – the linear coherent model, which our method can deal with easily.

Based on the geometric perspective discussed above, we propose a geometric gene expression biclustering framework that involves the following two steps. First, we detect the hyperplanes that exist in the gene expression data. Then we analyze whether a required pattern exists for the genes that lie in these hyperplanes.

A powerful technique for line detection in noisy 2-D images and for plane detection in noisy 3-D data called the Hough transform (HT) [30] is widely used in pattern recognition. The HT has been extensively studied in image processing and is well known to be robust against noise for line detection in poor quality images. This robustness is especially useful in microarray data analysis since the data are often heavily corrupted by noise. The method has recently been applied successfully to two and three-color microarray data analysis [31,32]. Interested readers are referred to the survey paper [33] on the properties and general applications of the HT.

However, it may be difficult to use the standard HT for more than 3 dimensions because of the large computational complexity and storage requirement. In this work, we use the Fast Hough transform (FHT) [34] as our plane detection algorithm since it gives considerable speedup and requires less storage than the conventional HT. The FHT has a very simple and efficient high-dimensional extension. Furthermore, the FHT uses a coarse-to-fine mechanism and provides good noise resistibility. In the following, we briefly discuss the basic principles of the FHT.

### Plane detection using the fast Hough transform

We use {*F*_0_, *F*_1_... *F*_*M*-1_} to denote the coordinates of *M *arrays. For each gene *j *{*j *= 1, 2... *N*}, the expression vector is given as [*F*_0_(*j*), *F*_1_(*j*), ..., *F*_*M*-1_(*j*)].

In a 2-D space, a line can be described by

(5) *y *= *mx *+ *c*,

where (*m*, *c*) are two parameters: the slope and the intercept of the line with *y *axis. However, a problem with the (*m*, *c*) parameterization of lines is its inability to describe vertical lines, i.e., m → ∞. Therefore, Eq. (5) is only used for lines with |*m*| ≤ 1. The second equation that swaps the roles of the *x *and *y *axes,

(6) *x *= *m*'*y *+ *c*'

is used for lines with |*m*| > 1. With |*m*| ≤ 1 and |*m*'| < 1, Eq. (2) and (3) describe all lines in a 2-D space without overlap. A similar method can be used to describe hyperplanes in a high dimensional space. In this paper, this parameterization method is used for our hyperplane detection algorithm.

Suppose that among all the observed data [*F*_0_(*j*), *F*_1_(*j*), ..., *F*_*M*-1_(*j*)], {*j *= 1, 2, ..., *N*}, there exists a target hyperplane described by the following equation

(7) $$F_{0}=\sum_{i=1}^{M-1}\beta_{i}F_{i}+\beta_{M},$$

where {*F*_0_, *F*_1_, ..., *F*_*M*-1_} are coordinates of points in observed data space and {*β*_1_, *β*_2_, ..., *β*_*M*_} are *M *parameters. Define a set Ω with all the indices of the genes that lies on this hyperplane. For each *j *∈ Ω, we have $F_{0}(j)=\sum_{i=1}^{M-1}\beta_{i}F_{i}(j)+\beta_{M}$. The inversion of (7) indicates that all these points on the target surface satisfy

(8) $$\begin{array}{cc} \sum_{i=1}^{M-1}F_{i}(j)\beta_{i}+\beta_{M}-F_{0}(j)=0 & \text{for all }j\in \Omega . \end{array}$$

We find that the parameters {*β*_1_, *β*_2_, ..., *β*_*M*_} are given by the intersection of many hyperplanes given by Eq. (8).

Suppose that we know the initial ranges of value {*β*_1_, *β*_2_, ..., *β*_*M*_} are centered at {*P*_1_, *P*_2_, ..., *P*_*M*_} and with half-length {*L*_1_, *L*_2_, ..., *L*_*M*_}. We can divide these ranges into very small "array accumulators" so that each array accumulator can determine a unique array of values {*β*_1_, *β*_2_, ..., *β*_*M*_} within the acceptable tolerance. According to Eq. (8), one feature point in the observed signal space is mapped into many points (e.g., hyperplanes) in the parameter space. An accumulator in the parameter space containing many mapped points (e.g., the intersection of many hyperplanes) reveals the potential feature of interest.

According to above analysis, the FHT-based plane detection method includes three parts. First, we need a hyperplane formulation as in Eq. (8). Second, we divide the parameter space into accumulators that is small enough so that the desired resolution is satisfied. Third, for the accumulators, let every point in the observed data vote for them. If the votes that an accumulator receives is more than a selected threshold, we detect a hyperplane in the observed data space as given by Eq. (7), where the values of {*β*_1_, *β*_2_, ..., *β*_*M*_} are given by the accumulator. Now we introduce each part of the algorithm in details.

#### Hyperplane formulation

The FHT does not use Eq. (8) directly. Suppose that we know the initial ranges of values {*β*_1_, *β*_2_, ..., *β*_*M*_} are centered at {*P*_1_, *P*_2_, ..., *P*_*M*_} and with half-length {*L*_1_, *L*_2_, ..., *L*_*M*_}. According to Eq. (8), we have

(9) $$\begin{array}{cc} \sum_{i=1}^{M-1}\frac{F_{i}(j)L_{i}}{W(j)L_{M}}\frac{\beta_{i}}{L_{i}}+\frac{\beta_{M}}{W(j)L_{M}}-\frac{F_{0}(j)}{W(j)L_{M}}=0 & \text{for all }j\in \Omega , \end{array}$$

where *W*(*j*) is a weighting scale used to ensure that $\sum_{i=1}^{M}a_{i}^{2}(j)=1$. Let $X_{i}=\frac{\beta_{i}}{L_{i}}$ (*i *= 1, ..., *M*), $\frac{F_{i}(j)L_{i}}{W(j)L_{M}}=a_{i}(j)$ (*i *= 1, ..., *M*-1), $a_{0}(j)=-\frac{F_{0}(j)}{W(j)L_{M}}$ and $a_{M}(j)=\frac{1}{W(j)}$, Eq. (9) can be rewritten as

(10) $$\begin{array}{cc} \sum_{i=1}^{M}a_{i}(j)X_{i}+a_{0}(j)=0 & \text{for all }j\in \Omega . \end{array}$$

In fact, it is not necessary for the dimension of the parameter space *X *to be equal to the dimension of observed signal, *M*. We use *k *to replace *M *for a more general expression

(11) $$\begin{array}{cc} \sum_{i=1}^{k}a_{i}(j)X_{i}+a_{0}(j)=0 & \text{for all }j\in \Omega , \end{array}$$

where *X*_*i *_is the *i*-th dimension of the parameter space. Each *a*_*i*_(*j*) is a function of observed feature points and is normalized such that $\sum_{i=1}^{k}a_{i}^{2}(j)=1$. The initial range for each *X*_*i *_is an interval of length 2, with center at *P*_*i*_/*L*_*i*_. All these ranges comprise a *hypercube *in the parameter space (*X*_1_, ...., *X*_*k*_).

#### Vote counting scheme

As mentioned before, every point in the observed data votes for supporting accumulators. We know that each accumulator corresponds to a group of range values of (*X*_1_, *X*_2_, ..., *X*_*M*_). For each point *j *in the observed data, if $\sum_{i=1}^{k}a_{i}(j)X_{i}+a_{0}(j)=0$ can be satisfied when the values of (*X*_1_, *X*_2_, ..., *X*_*M*_) lie in this accumulator, and it will give a vote to this accumulator. An accumulator receiving votes more than a threshold reveals a corresponding hyperplane in the observed data space.

So, to determine whether an accumulator received a vote from a point *j *in observed signals, we only need to determine whether a hypercube (accumulator) intersect with a particular hyperplane $\sum_{i=1}^{k}a_{i}(j)X_{i}+a_{0}(j)=0$. We can use a simpler conservative test to check whether the hyperplane intersects the hypercube's circumscribing hypersphere. Assume the center of the accumulator is at [*C*_1_, ..., *C*_*k *_] and *r *is the radius of the hypersphere. We check whether

(12) $$a_{0}(j)+\sum_{i=1}^{k}a_{i}(j)C_{i}\leq r$$

If Eq. (12) is satisfied, gene *j *will give a vote to the corresponding accumulator.

#### K-tree representation

For simplicity, we have assumed above that the parameter space was directly divided into very small accumulators. Actually, this is not necessary. The FHT algorithm recursively divides the parameter space into hypercubes from low to high resolutions. It performs the subdivision and the subsequent "vote counting" is done only in hypercubes with votes exceeding a selected threshold. This hierarchical approach leads to a significant reduction in both computational time and storage space compared to the conventional HT.

For the FHT, we represent the parameter space as a nested hierarchy hypercube. We can associate a *k*-tree with the representation. The root node of the tree corresponds to a hypercube centered at vector C_0 _with side-length *S*_0_. Each node of the tree has 2^*k *^children arising when that node's hypercube is halved along each of its *k *dimensions. Each child has a child index, a vector **b **= [*b*_1_, ..., *b*_*k*_], where each *b*_*i *_is - 1 or 1. The child index is interpreted as follows: if a node at level *l *of the tree has center **C**_*l*_, then the center of its child node with index [*b*_1_, ..., *b*_*k*_] is

(13) $$C_{l}+\frac{S_{l+1}}{2}+b,$$

where S_*l*+1 _is the side length of the child at level *l*+1 and *S*_*l*+1 _= *S*_*l*_/2.

Since we use a coarse-to-fine mechanism, for each accumulator at different levels we need to make a test using Eq. (12). For an accumulator of level *l*, the radius of its circumscribing hypersphere *r *is equal to $\sqrt{k}S_{l}/2$. Based on the *K*-tree structure, an incremental formula can be used to calculate the left part of Eq. (12). If we divide the left part of Eq. (12) by *S*_*l*_, the normalized distance can be computed incrementally for a child node at level *l *with child index [*b*_1_, ..., *b*_*k*_] as follows,

(14) $$R_{0}(j)=\frac{a_{0}(j)}{S_{0}}+\sum_{i=1}^{k}a_{i}(j)\cdot \frac{C_{0}(i)}{S_{0}};$$

(15) $$R_{l}(j)=2R_{l-1}(j)+\frac{1}{2}\sum_{i=1}^{k}a_{i}(j)b_{i}.$$

Test of Eq. (12) can now be expressed as: for the gene *j *and a child node with child index [*b*_1_, ..., *b*_*k*_] at level *l*, if

(16) $$|R_{l}(j)|\leq \sqrt{k}/2,$$

gene *j *will generate a vote for this child node.

According to the above analysis, the FHT is a mapping from an observed *data space *into a *parameter space*. Each feature point in the data space generates "votes" for a set of sub-areas (hypercubes) in the parameter space. A sub-area in the parameter space that receives many votes reveals the feature of interest. The FHT algorithm recursively divides the parameter space into hypercubes from low to high resolutions. It performs the subdivision and the subsequent "vote counting" is done only in hypercubes with the number of votes exceeding a selected threshold. A hypercube with acceptable resolution and with votes exceeding a selected threshold indicate a detected hyperplane in the observed data.

### The proposed geometric biclustering algorithm and parameter selection

To summarize, when given a set of genes expression data [*F*_0_(*j*), *F*_1_(*j*), ..., *F*_*M*-1_(*j*)], *j *= 1, 2,..., *N *under diverse experimental conditions, our geometric biclustering algorithm can be summarized as follows:

Parameters that need to be predetermined:

(1) The minimum votes count "*T*" as threshold and the desired finest resolution "*q*".

(2) A transformation that maps gene expression data [*F*_0_(*j*), *F*_1_(*j*), ..., *F*_*M*-1_(*j*)] into a hyperplane in the parameter space represented by $\sum_{i=1}^{k}a_{i}(j)X_{i}+a_{0}(j)=0$ for *j *= 1,2,⋯,*N*. Based on the transformation, determine the initial bound of each *X*_*i *_and the root hypercube.

Biclustering procedure:

(1) Map gene expression data onto the parameter space.

(2) Compute the initial normalized distance from the hyperplane to the root node and perform the voting procedure for the root node. For each gene, if Eq. (16) is satisfied, add one to the vote count of the root node. If the vote count for root node is larger than the threshold T and the resolution is coarser than *q*, subdivide the root node into the *K*-tree child nodes.

(3) Vote for each child node and subdivide them if needed. A similar vote-and-subdivide mechanism is performed for each new node until no new node appears.

(4) When there is no node with resolution equal to *q *and the vote count larger than T, record the node with the finest resolution. This is the most probable solution. When there are several nodes with resolution equal to *q *and vote counts larger than T, collect the planes associated with these nodes that have the same genes into a bundle.

(5) For each bundle of hyperplanes, check the common conditions (variables) and compare the hyperplanes with the models corresponding to different types of biclusters. A bundle of hyperplanes that are not consistent with any patterns in Fig. 2 or the corresponding bicluster covers too few samples will be discarded. If the bundle survives this process, it will be output as a bicluster. Repeat this step until all bundles are processed.

In the procedure above, there are two parameters: minimum vote count "T" and the desired finest resolution "*q*". The minimum vote count "T" denotes the minimum number of genes in a bicluster. T depends on the experiment objective and may be selected by the user. For example, the minimum may be 4, that is, a bicluster must contain at least 4 genes. The desired finest resolution "*q*" depends on the variance of noise in the data. For a perfect bicluster (for example, a perfect constant bicluster where all values are equal), "*q*" can be arbitrarily large, that is, one can use an arbitrarily fine resolution. However, in practical applications, perfect biclusters are rarely found and "*q*" reflects how much noise (or inconsistency) is permitted in the detected biclusters. If we wish to detect strongly coherent biclusters (i.e., near perfect bicluster with very little noise), *q *should be set to a large number. Smaller *q *can be used to detect biclusters that exhibit more inconsistency due to noise. In general, larger *q *results in biclusters of smaller size.

In many situations, one has no knowledge about the noise in the data. An appropriate range of *q *can be determined experimentally to return meaningful biclusters. Recall that the FHT uses a coarse-to-fine mechanism. At coarse resolution, there are fewer accumulator cells and the number of hyperplanes detected is small. At finer resolution, there are more accumulator cells. However, in this case the accumulator cells are also smaller and it is more difficult for a feature point to generate a hit. Many accumulators therefore cannot gain enough votes (exceeding the threshold) to ensure the existence of the corresponding hyperplane. So, if *q *is set too large or too small, fewer hyperplanes will be detected. Hence, the range of *q *can be chosen to be one that returns a reasonably large number of hyperplanes.

### Computational complexity

For FHT, the following theorem from [34] limits the computational complexity. The "thin tree" property resulting from the theorem guarantees that the complexity of the FHT does not go beyond the bound due to the chosen *q*.

*Theorem *[34]:

Assume that all M hyperplanes in the parameter space intersect at a single point C and that they are uniformly distributed in orientation. Given a minimum vote threshold T, the number of hypercubes of size q that can receive T or more votes is less than some number K that does not depend on q", where

$$\begin{array}{ccc} K=4K_{1}^{2} & \text{with} & K_{1}=\text{ceil}\left(\frac{\sqrt{2}}{2}/\tan\left(\frac{\pi T}{2M}\right)\right)+1 \end{array}$$

The FHT algorithm is highly parallel. As shown, the processing for the hypercubes or accumulators is independent of each other. Furthermore, the intersection test for a hyperplane does not depend on that of other hyperplanes. Actually, in our implementation, some simple multi-processing optimization, such as OpenMP or OpenMPI library, can achieve a high level of speedup.

In the above discussion, we assume that all the possible linear hyperlanes are to be detected using the FHT. In practice, detecting a small portion of hyperplanes is already enough for our biclustering algorithm. For example, in a dataset [*F*_0_(*j*), *F*_1_(*j*), ..., *F*_*M*-1_(*j*)], {*j *= 1, 2, ..., *N*, using Equation $F_{0}=\sum_{i=1}^{M-1}\beta_{i}F_{i}+\beta_{M}$ we can find all the biclusters covering *F*_0_. However, using Equation $F_{0}=\sum_{i=1}^{M-1}\beta_{i}F_{i}+\beta_{M}$ with *β*_*i *_= 0 *or t *and *t *is a scale, i.e., a constraint that requires all the non-zero gradients to be equal, we can also find all the bicusters covering *F*_0_. The second equation can significantly lower the comptational burden^1^. Another optimization direction is to take advantage of the property of the gene expression data. Since the gene expression data values are distributed in the range of [-5 5], the hyperplanes $\sum_{i=1}^{M}0.3X_{i}+20=0$ or $60X_{1}+\sum_{i=2}^{M}0.3X_{i}+0.2=0$ do not have any practical significance and can be disregarded. So scanning the dataset to determine the range of hyperplane parameters before biclustering can significantly lower the computational burden.

In certain special cases, we can simplify the problem according to the bicluster model. For example, if we extract biclusters of constant row, we only need to detect all the hyperplanes with $\sum_{i=1}^{M}a_{i}X_{i}=0$, *a*_*i *_= 0, 1 or -1, and if we extract multiplicative biclusters, we only need to detect those hyperplanes without intercept.

In term of CPU time, our algorithm is computationally intensive in its un-optimized general form. Based on the complexity of the FHT, the computational demands of the proposed biclustering algorithm depends on how many biclusters exist in the dataset. To give an indication of the computational cost, we run the un-optimized algorithm on a small test dataset on a personal computer (Linux OS with 2.0 G Intel Core 2 Duo processor and 1 GB memory) and record the CPU time.

We randomly select 16 conditions in Human Lymphoma Dataset to produce a 4026 × 16 matrix. The CPU time for over 800 biclusters is 1953 seconds (32.55 minutes). We can adjust the parameters to exclude small and noisy biclusters and reduce the computing time. For example, the CPU time reduces to 397 seconds (6.62 minutes) if we discard biclusters with less than 8 conditions.

For larger dataset, we need to run our algorithm on a computer cluster. For the entire 4026 × 96 Human Lymphoma Dataset, we run our algorithm on a computer cluster of 8 nodes with 2 processors each and it takes about 22 hours. Hence, the proposed algorithm is very time-consuming for large datasets if we search through the entire high-dimensional Hough space to obtain the optimal solution and detect all possible additive and multiplicative coherent patterns in the data.

The computing time can be substantially reduced if we allow the solution to the sub-optimal. For example, we can divide 96 conditions into 6 sets with 16 conditions in each set. Then, only 39.7 (6 × 6.62) minutes are needed on Linux computer described above for the biclustering process. The biclusters from the 6 sets can then be combined. Such a strategy has already been used in [18]. We can also consider two conditions at a time and then combine sub-biclusters gradually to form large ones [32].

## Abbreviations

GO: Gene Ontology, 2D: Two dimensional, 3D: Three dimensional, NP: Non-deterministic polynomial time, HT: Hough transform.

## Authors' contributions

XG worked on the hyperplane modeling, implementation and experimental analysis when he was a Ph.D. student at City University of Hong Kong. AWCL proposed the geometric perspective for biclustering, problem formulation, and algorithm design. Both XG and AWCL contributed equally to this work and should be considered as joint first author. HY initiated the project and worked on the Hough transform. All authors read and approved the final manuscript.

## Note

^1 ^This method is easy to implement by only testing the hyperplane/accumulator with equal non-zero gradients. Assume there are *t *subranges for each *β*_*i *_. If we do not consider the coarse-to-fine optimization of FHT, the first equation need to process *t *^*M *^accumulator while the second equation only need to process about *t*^2^*2^*M*-1^. In the case of *t *= 5, *M *= 40, the computational burden of the second scheme is 1.5*10^-15 ^times that of the first scheme.

## Supplementary Material

Additional file 1 Information for additive biclusters detection on the Human Lymphoma Dataset. The parameters used in the proposed biclustering algorithm for the Human Lymphoma Dataset are given. Click here for file

Additional file 2 All detected biclusters. A list of all biclusters with 1 showing corresponding genes/arrays covered by the bicluster while 0 is the contrary. Click here for file

Additional file 3 GO annotation of six selected biclusters. The expression heat map and GO annotation table of six biclusters are given here. Click here for file

Additional file 4 All detected biclusters with full data. All the detected biclusters with full data are given here. Click here for file

Additional file 5 A bicluster of linear coherent values in the lymphoma dataset. A full size image showing the linear coherent bicluster detected. Click here for file
