# Supplementary material for: Discovering biclusters in gene expression data based on high-dimensional linear geometries
Source: BMC Bioinformatics. 2008 Apr 23;9:209. doi: 10.1186/1471-2105-9-209 (PMC2386490; doi:10.1186/1471-2105-9-209)
Supplement: Additional file 1 — Information for additive biclusters detection on the Human Lymphoma Dataset. The parameters used in the proposed biclustering algorithm for the Human Lymphoma Dataset are given. [file 1471-2105-9-209-S1.doc]

Supplementary information for:

**Discovering Biclusters in Gene Expression Data based on High-Dimensional Linear Geometries**

Information for additive biclusters detection on Human Lymphoma Dataset**:**

**Dataset**: Human Lymphoma Dataset (Alizadeh et al. Nature 2000, 403(6769):503-511)

**Parameters**:

1. The threshold for the number of genes that makes a significant bicluster: 450

2. The threshold for the number of conditions that makes a significant bicluster: 8

3. The noise bound: 0.65

4. To judge whether a bicluster are GO significant, we adopted the following standard:

First, we use FuncAssociate (<http://llama.med.harvard.edu/cgi/func/funcassociate>) to report enriched function or process. Only when a function or process shared by more than 100 genes in a bicluster is available and is GO-enriched, the bicluster is regarded as significant.

**Results**: In our experiment, more than 600 biclusters are detected.

- A list of all biclusters with 1 showing corresponding genes/arrays covered by the bicluster while 0 is the contrary is in: biclu_list.txt [see Additional file 2]
- We selected 6 biclusters as an example for GO annotation. The expression figure and GO annotation table is in: biclu_example.doc [see Additional file 3]
- All the biclusters with full data are in: biclu_result.zip [see Additional file 4]
