# Supplementary figures and images for: Discovering biclusters in gene expression data based on high-dimensional linear geometries
Source: BMC Bioinformatics. 2008 Apr 23;9:209. doi: 10.1186/1471-2105-9-209 (PMC2386490; doi:10.1186/1471-2105-9-209)

Bi_1


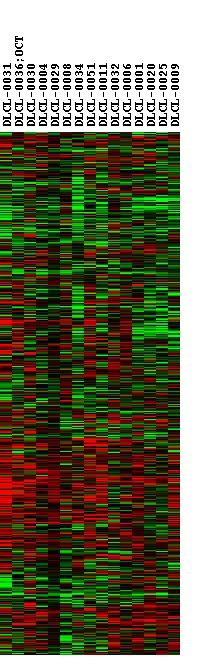


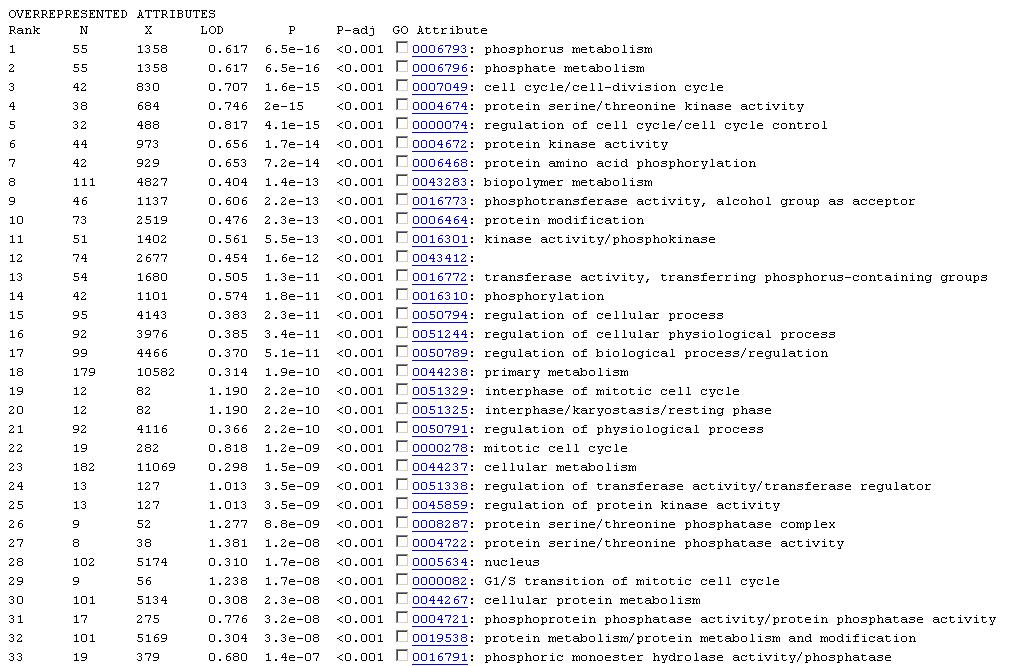


bi_7


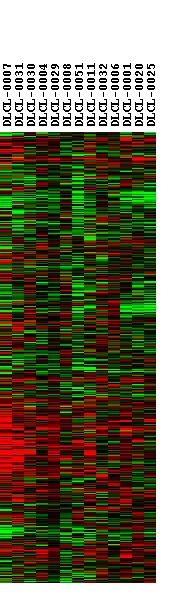

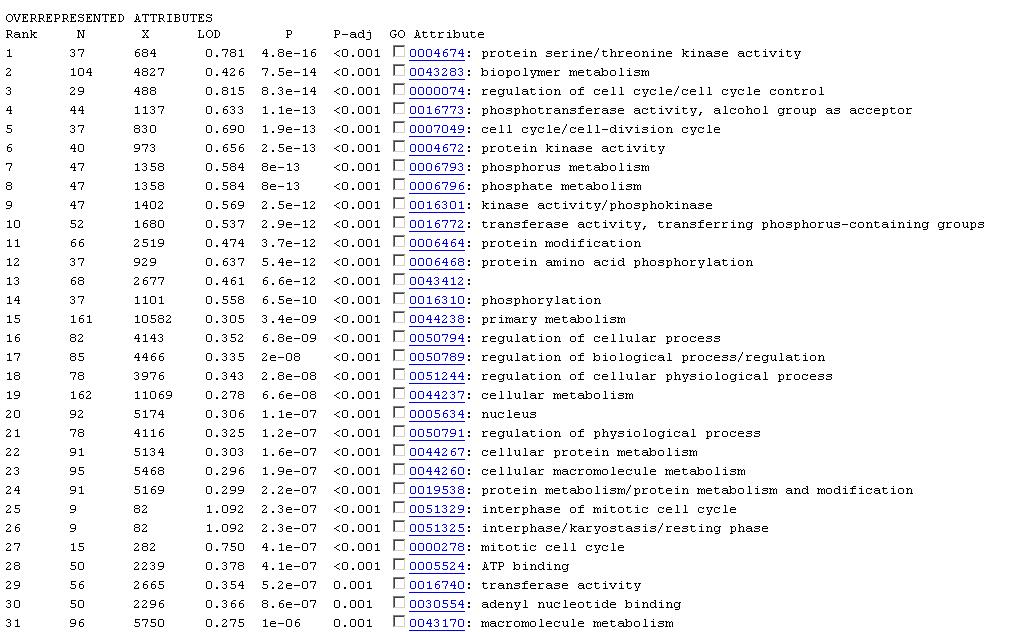


bi_524


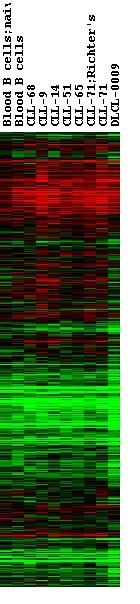


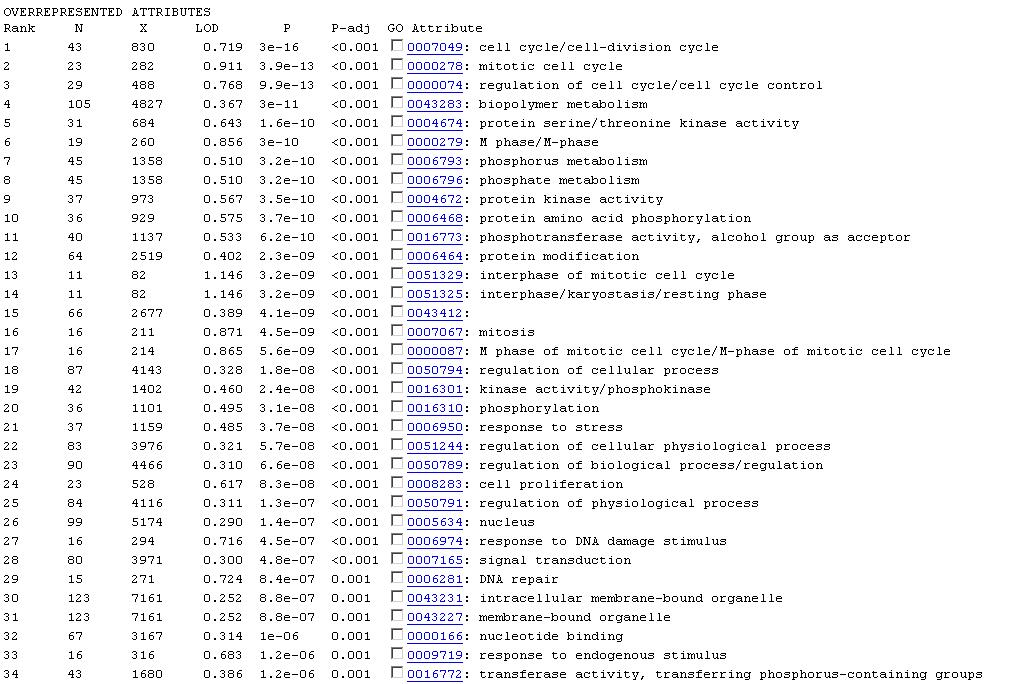


bi_548


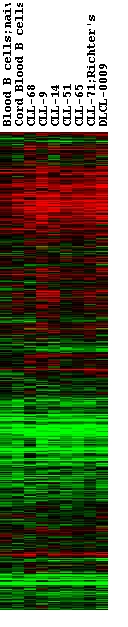

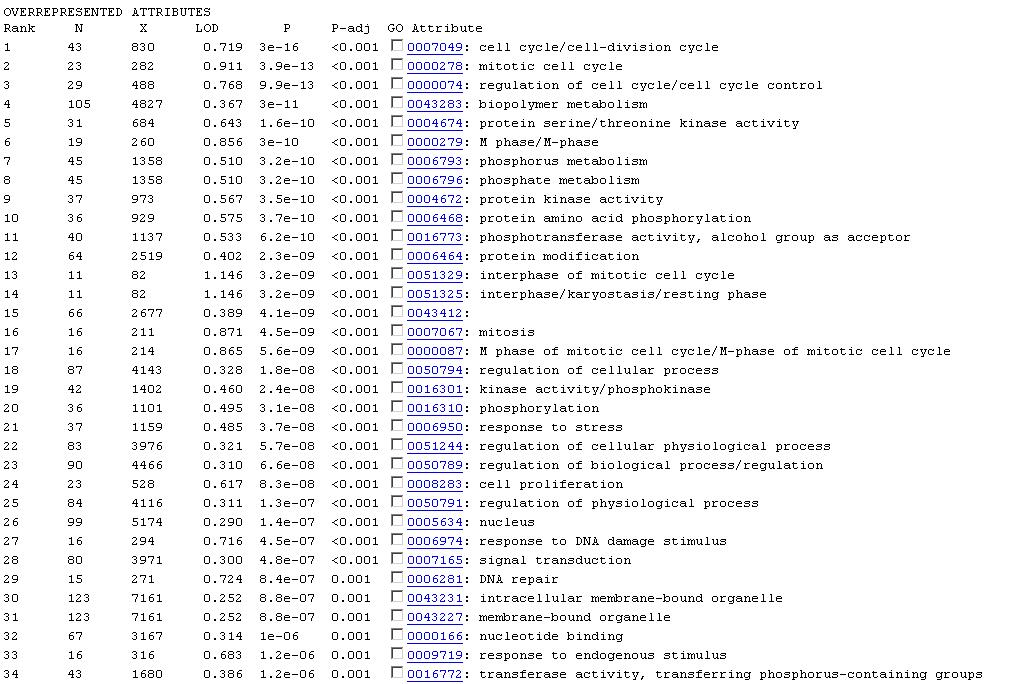


bi_600


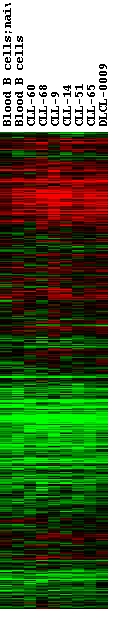

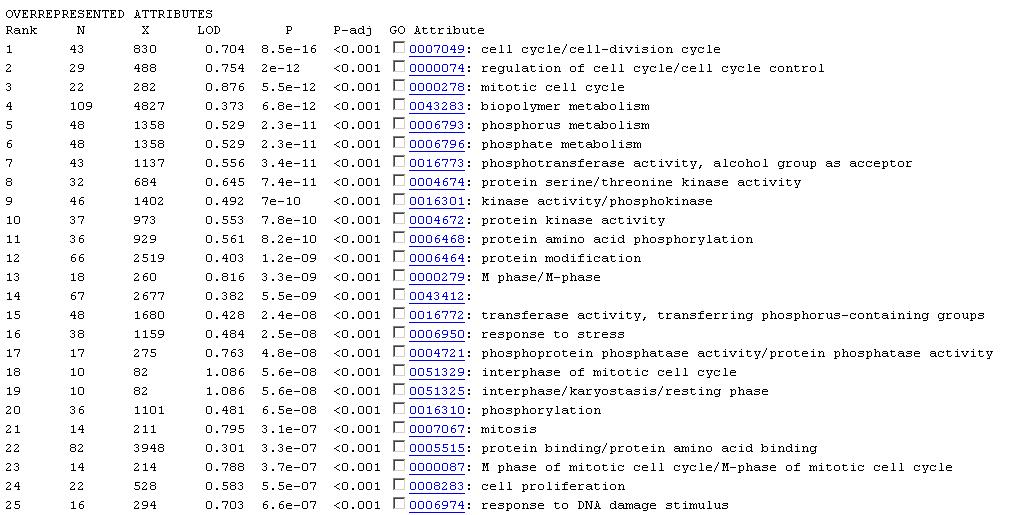


bi_605


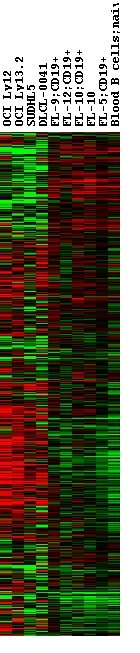

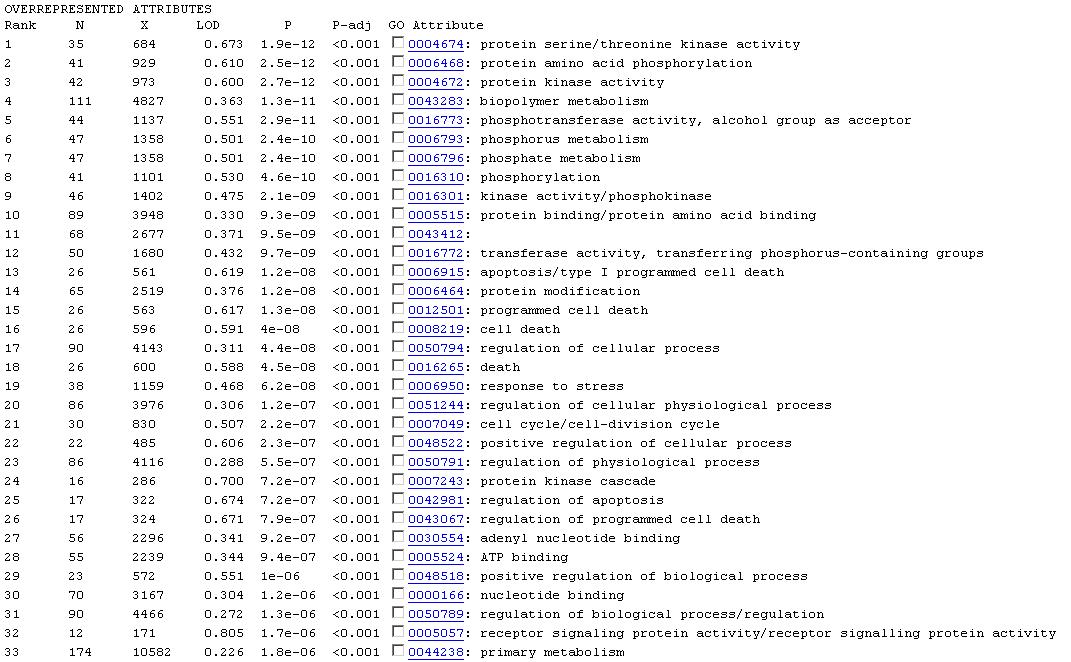

Supplement: Additional file 3 — GO annotation of six selected biclusters. The expression heat map and GO annotation table of six biclusters are given here. [file 1471-2105-9-209-S3.doc]
