# Supplementary material for: Discovering biclusters in gene expression data based on high-dimensional linear geometries
Source: BMC Bioinformatics. 2008 Apr 23;9:209. doi: 10.1186/1471-2105-9-209 (PMC2386490; doi:10.1186/1471-2105-9-209)
Supplement: Additional file 5 — A bicluster of linear coherent values in the lymphoma dataset. A full size image showing the linear coherent bicluster detected. [file 1471-2105-9-209-S5.pdf]

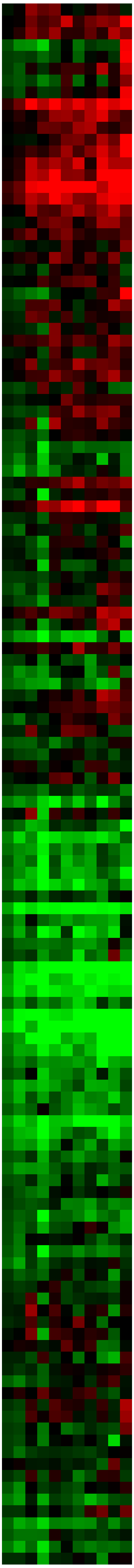

CLL-39
CLL-52
CLL-13
CLL-71
CLL-71;Richters
CLL-65
CLL-51
CLL-14
CLL-9
CLL-68
CLL-60
Unknown UG Hs.72045 ESTs; Clone=1268758
Unknown; Clone=703791
*Unknown UG Hs.132739 Homo sapiens HIC protein mRNA, complete cds; Clone=1367394
*High mobility group nonhistone chromosomal protein isoforms I and Y; Clone=344413
*MEF2A=MADS/MEF2-family transcription factor=myocyte enhancer-binding factor 2A; Clone=298320
*inducible protein; Clone=1290386
*CD22; Clone=1234404
*CD79A=BCR alpha chain=mb-1; Clone=115281
*lyn=tyrosine kinase; Clone=193913
Unknown UG Hs.133947 ESTs; Clone=1672025
Unknown UG Hs.140628 ESTs; Clone=1339337
*KIAA0226; Clone=1336149
Unknown; Clone=703659
*p27kip1=cyclin kinase inhibitor; Clone=627509
Integrin, alpha V vitronectin receptor, alpha polypeptide, antigen CD51; Clone=301551
*Unknown; Clone=1338510
Unknown; Clone=1368868
*Similar to KIAA0191= C2H2 zinc-finger at its N-terminal region; Clone=1351962
Unknown UG Hs.193977 ESTs, Weakly similar to neuronal thread protein AD7c-NTP [H.sapiens]; Clone=826617
Unknown UG Hs.124344 ESTs, Weakly similar to !!!! ALU CLASS C WARNING ENTRY !!!! [H.sapiens]; Clone=1352894
*KIAA0128=Similar to septin; Clone=293107
Unknown UG Hs.231798 ESTs; Clone=827169
*Unknown UG Hs.172825 Homo sapiens mRNA for KIAA1037 protein, partial cds; Clone=1367802
Unknown UG Hs.58383 ESTs; Clone=1353372
Unknown UG Hs.25600 ESTs; Clone=1339070
Pig12=p53-inducible gene; Clone=491213
HU-PP-1=protein-tyrosine phosphatase; Clone=825442
Vasopressin activated calcium mobilizing receptor-like protein; Clone=1289255
*Unknown UG Hs.120975 ESTs, Weakly similar to define not available 5262644 [H.sapiens]; Clone=1185293
TRANCE=RANKL=Osteoprotegerin ligand=TNF-related ligand; Clone=825287
*BRCA2 region EST-1; Clone=429238
Unknown; Clone=1370845
*Cyclin H; Clone=1320371
Unknown; Clone=1339998
*BAP135=Bruton's tyrosine kinase-associated protein-135; Clone=1286217
Unknown UG Hs.11463 ESTs, Weakly similar to define not available 4691541 [H.sapiens]; Clone=1320421
*40 kDa protein kinase related to rat ERK2; Clone=1339107
Unknown UG Hs.144097 ESTs; Clone=1241900
Unknown UG Hs.123283 EST; Clone=1352190
Unknown UG Hs.105252 ESTs; Clone=1285850
*Similar to p130 = RB related protein; Clone=1283405
Unknown; Clone=1355369
Unknown; Clone=1355689
*Unknown UG Hs.36672 ESTs; Clone=1269317
Unknown UG Hs.172550 polypyrimidine tract binding protein heterogeneous nuclear ribonucleoprotein I; Clone=1371619
Unknown UG Hs.229233 EST, Moderately similar to putative p150 [H.sapiens]; Clone=1270949
Smad4=DPC4=Homologue of Mothers Against Decapentaplegic MAD=required for TGF beta signaling=tumor suppressor in pancreatic cancer; Clone=774619
*Glucocorticoid receptor; Clone=1283110
branched chain aminotransferase precursor BCATm; Clone=43773
*Unknown UG Hs.214455 ESTs, Weakly similar to HNK-1 sulfotransferase [R.norvegicus]; Clone=1371202
*histone H2A.X; Clone=1418925
Unknown UG Hs.113759 ESTs; Clone=1353479
Unknown; Clone=683972
Unknown UG Hs.33054 ESTs; Clone=685710
Unknown UG Hs.187478 ESTs; Clone=1372156
*Unknown UG Hs.180660 EST; Clone=1336424
*Unknown UG Hs.123376 EST; Clone=1337710
*VASP=Vasodilator-stimulated phosphoprotein=focal adhesion and microfilament-associated protein; Clone=1371613
Unknown; Clone=LC17553
*OX-40; Clone=384692
Autocrine motility factor receptor; Clone=1301814
*CD102=ICAM-2=intercellular adhesion molecule 2; Clone=1235170
*FES=FPS=Tyrosine protein kinase; Clone=1286005
Similar to Z73581 ORF YPL225w [Saccharomyces cerevisiae]; Clone=1303644
Unknown UG Hs.171847 ESTs; Clone=1339063
*Unknown UG Hs.191322 ESTs; Clone=1369117
*Protein phosphatase 2 formerly 2A, regulatory subunit B PR 52, alpha isoform; Clone=704413
*fvt1=Follicular lymphoma variant translocation 1=putatively secreted protein translocated in t2;18p11;q21 follicular lymphoma and CLL; Clone=814260
Unknown UG Hs.120363 ESTs; Clone=1317045
Similar to putative outer mitochondrial membrane 34 kDa translocase hTOM34; Clone=1289116
Unknown; Clone=1355850
*Protein phosphatase 2 formerly 2A, catalytic subunit, alpha isoform; Clone=141688
*Protein phosphatase 2 formerly 2A, catalytic subunit, alpha isoform; Clone=566084
CSF-1=Macrophage colony stimulating factor-1; Clone=1371832
Unknown; Clone=LC18602
*Signal recognition particle 72 SRP72 with overlapping cam kinase II isoform; Clone=1234212
*G protein gamma-10 subunit; Clone=196333
Rat translocon-associated protein delta homolog; Clone=1354406
Unknown UG Hs.105492 ESTs; Clone=826629
*progesterone receptor-associated p48 protein=putative tumor suppressor SNC6; Clone=210887
*dUTP pyrophosphatase=deoxyuridine triphosphatase DUT; Clone=703799
Unknown; Clone=1340158
cell cycle protein p38-2G4 homolog hG4-1; Clone=1307238
*Tumor Associated Antigen L6; Clone=78113
*SLAP=src-like adapter protein; Clone=815774
*aurora/IPL1-related kinase; Clone=1357548
Unknown; Clone=1357921
*DNA cytosine-5--methyltransferase; Clone=45941
*DNA cytosine-5--methyltransferase; Clone=1320361
*protein phosphatase 2A 74 kDa regulatory subunit delta or B subunit; Clone=429029
Unknown; Clone=1355881
*CLPP=ATP-dependent Clp proteinase; Clone=279713
cdc25C=M-phase inducer phosphatase 3; Clone=121066
*Smad6=JV15-1=Homologue of Mothers Against Decapentaplegic MAD=negative regulator of TGF beta signaling; Clone=429356
*IMP inosine monophosphate dehydrogenase 2; Clone=814556
*MAPKAP kinase 3pK; Clone=296513
*Casein kinase II alpha chain; Clone=1286561
*MLF2=myelodysplasia/myeloid leukemia factor 2; Clone=810743
*Host cell factor-1=VP16 transactivator interacting protein; Clone=1234225
*polyADP-ribose glycohydrolase; Clone=1309002
BAT2=large proline-rich protein=MHC class III histocompatibility antigen HLA-B-associated transcript 2; Clone=746038
*KIAA0078=Similar to double-straded break repair protein RAD21; Clone=1320406
*PPP1CB=Protein phosphatase 1, catalytic subunit, beta isoform; Clone=485729
*BRCA2=Mutated in breast and ovarian cancer; Clone=1340447
BAP31= preferentially associated with membrane IgD but only weakly with membrane IgM; Clone=1242061
*PKA-C-ALPHA=cAMP-dependent protein kinase, alpha-catalytic subunit; Clone=245232
*Glycogen synthase kinase 3 beta; Clone=1183990
*PMS4=DNA mismatch repair protein; Clone=758206
*ribosomal protein L5 pseudogene; Clone=1283900
mss4=Zn2+ binding protein/guanine nucleotide exchange factor; Clone=230857
Acidic ribosomal phosphoprotein P2; Clone=1340095
Similar to mitochondrial 3-ketoacyl-CoA thiolase beta-subunit of trifunctional protein; Clone=1335102
Unknown UG Hs.120239 ESTs; Clone=1339160
Unknown UG Hs.171228 Homo sapiens mRNA for KIAA1040 protein, partial cds; Clone=825350
Unknown UG Hs.219237 ESTs, Highly similar to !!!! ALU SUBFAMILY SX WARNING ENTRY !!!! [H.sapiens]; Clone=1372254
Unknown UG Hs.5735 ESTs; Clone=1283932
*acid finger protein; Clone=755176
*Unknown UG Hs.122983 ESTs; Clone=1304422
*MHC Class I=HLA-C4; Clone=810142
*MHC Class I=HLA-B27; Clone=769753
*MHC Class I=HLA-A2; Clone=203527
*Unknown UG Hs.151945 CAAX box 1; Clone=704919
MNB=homologue of Drosophila minibrain=serine/threonine kinase; Clone=1234239
PISSLRE = novel cdk; Clone=1351649
*BAK=BCL-2 family member; Clone=235938
Unknown; Clone=1355753
CYR61=gig1=growth-factor inducible immediate early gene product; Clone=486700
*FCERI=Fc epsilon receptor gamma chain=High affinity immunoglobulin epsilon receptor gamma-subunit precursor; Clone=145932
Unknown; Clone=1672001
*SMRT=silencing mediator of retinoid and thyroid hormone action=corepressor; Clone=723911
Unknown UG Hs.173945 ESTs, Moderately similar to !!!! ALU CLASS A WARNING ENTRY !!!! [H.sapiens]; Clone=711452
Nonmusclemyosinheavychain;Clone=1302180
